# The Airway Microbiome at Birth

**DOI:** 10.1038/srep31023

**Published:** 2016-08-04

**Authors:** Charitharth Vivek Lal, Colm Travers, Zubair H. Aghai, Peter Eipers, Tamas Jilling, Brian Halloran, Waldemar A. Carlo, Jordan Keeley, Gabriel Rezonzew, Ranjit Kumar, Casey Morrow, Vineet Bhandari, Namasivayam Ambalavanan

**Affiliations:** 1 Division of Neonatology, Department of Pediatrics, University of Alabama at Birmingham, AL, USA; 2 Translational Research in Normal and Disordered Development Program (TReNDD) University of Alabama at Birmingham, AL, USA; 3 Program in Protease and Matrix Biology, University of Alabama at Birmingham, AL, USA; 4 Department of Pediatrics, Thomas Jefferson University/Nemours, Philadelphia, PA, USA; 5 Department of Cell, Developmental and Integrative Biology, University of Alabama at Birmingham, AL, USA; 6 Center for Clinical and Translational Sciences, University of Alabama at Birmingham, AL, USA; 7 Department of Pediatrics, Drexel University College of Medicine, Philadelphia, PA, USA.

## Abstract

Alterations of pulmonary microbiome have been recognized in multiple respiratory disorders. It is critically important to ascertain if an airway microbiome exists at birth and if so, whether it is associated with subsequent lung disease. We found an established diverse and similar airway microbiome at birth in both preterm and term infants, which was more diverse and different from that of older preterm infants with established chronic lung disease (bronchopulmonary dysplasia). Consistent temporal dysbiotic changes in the airway microbiome were seen from birth to the development of bronchopulmonary dysplasia in extremely preterm infants. Genus *Lactobacillus* was decreased at birth in infants with chorioamnionitis and in preterm infants who subsequently went on to develop lung disease. Our results, taken together with previous literature indicating a placental and amniotic fluid microbiome, suggest fetal acquisition of an airway microbiome. We speculate that the early airway microbiome may prime the developing pulmonary immune system, and dysbiosis in its development may set the stage for subsequent lung disease.

Microbiome analysis based on sequencing of 16S ribosomal RNA genes characterizes bacterial species present both in terms of their identities and relative abundance in contrast to traditional microbiological approaches that are restricted to analyzing species that are readily cultured^1^,^2^. The study of the human lung microbiome in the context of pulmonary health and disease is an area of emerging research^3^ but it is unknown as to when the respiratory microbiome is established, and whether the airways harbor a microbiome soon after birth. Evidence of pathogenic roles for specific alterations in airway microbiota is strongest in lung diseases such as cystic fibrosis, chronic obstructive pulmonary disease (COPD), and asthma but it is not clear how the airway microbiome is initially established in newborn infants^3^,^4^. Extremely premature infants are born with developmentally immature lungs that are susceptible to multiple injuries, which interfere with pulmonary alveolar and vascular development leading to bronchopulmonary dysplasia (BPD). It has been speculated that development of BPD is mediated at least in part by inflammation secondary to airway infections, and multiple studies have shown associations between lung inflammatory markers and BPD pathogenesis^5^,^6^,^7^,^8^. Yet the direction of causality between airway injury during development and respiratory colonization with microorganisms remains unsettled. Should a disordered airway microbiome prove to be involved in the pathogenesis of disease, it will be of immediate interest to attempt to develop novel therapeutic interventions. We hypothesized that an airway microbiome is present at birth, and that the airway microbiota at birth would differ between extremely low birth weight (ELBW) preterm infants (<1000 g birth weight) and full term (FT) infants, and would change over time in preterm infants who go on to develop BPD. We also hypothesized that early (at birth) airway microbiota would differ in ELBW infants who later go on to develop BPD from the ones who are resilient to the disease. We conducted the study initially at the University of Alabama at Birmingham (UAB) Regional Neonatal Intensive Care Unit (RNICU) and then confirmed our findings using a validation cohort from a different center in Philadelphia. In this study, we found a diverse airway microbiome at birth with prognostic potential that becomes dysregulated during development of BPD.

## Results

The demographics and clinical characteristics of enrolled patients in the Discovery Cohort are shown in Table 1. Based on their pulmonary outcomes, the 23 ELBW infants were further categorized as the ones who did not develop BPD (BPD Resistant, n = 13) or the ones who later developed BPD (BPD Predisposed, n = 10) at 36 weeks’ postmenstrual age (PMA). The demographics and clinical characteristics of enrolled ELBW patients from UAB are shown in Table 2.

The validation cohort was also categorized based on their pulmonary outcomes, into infants who did not go on to develop BPD (BPD Resistant, n = 7) or the ones who later developed BPD (BPD Predisposed, n = 7). The demographics and clinical characteristics of enrolled ELBW patients for the validation cohort are shown in Table 3.

To determine the temporal changes in airway microbiome, we collected serial tracheal aspirates (TA) at various time points from birth until the development of BPD in 5 infants from the discovery cohort.

### The airways of ELBW and FT infants have a distinct microbiota even at birth

**Figure 1:**
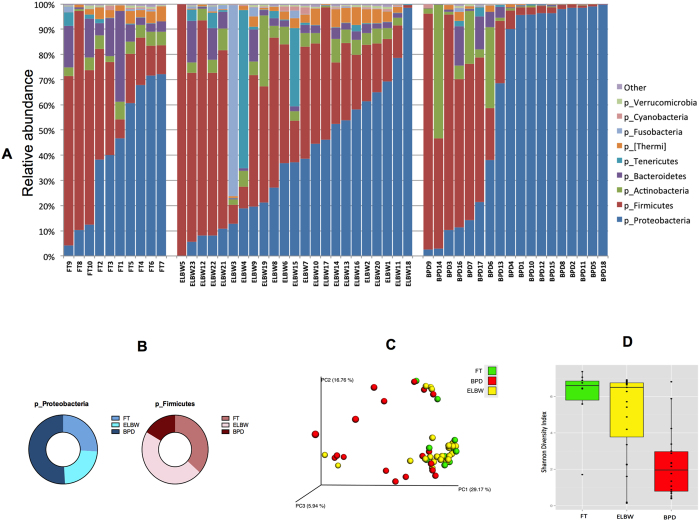
Comparison of lung microbiota of newborn infants and patients with BPD. (**A**) Bar graph depicting the relative abundance of most commonly encountered bacterial phyla between FT, ELBW and BPD infants. (**B**) Compared to newborn postmenstrual age matched FT infants and ELBW infants, infants with BPD have increased *Proteobacteria* and decreased *Firmicutes* and *Fusobacteria*. (**C**) Principal coordinates analysis ‘PCoA’ plot (beta diversity) demonstrating unweighted UniFrac distance between samples with sample points colored for ELBW, FT or BPD infants. Samples that are clustered closely together are considered to share a larger proportion of the phylogenetic tree in comparison to samples that are more separated. ELBW and FT infants have similar beta diversity, which is very different from the beta diversity of BPD infants. (**D**) Shannon diversity index depicting less microbial alpha diversity in infants with BPD compared to FT and ELBW infants.

**Figure 2:**
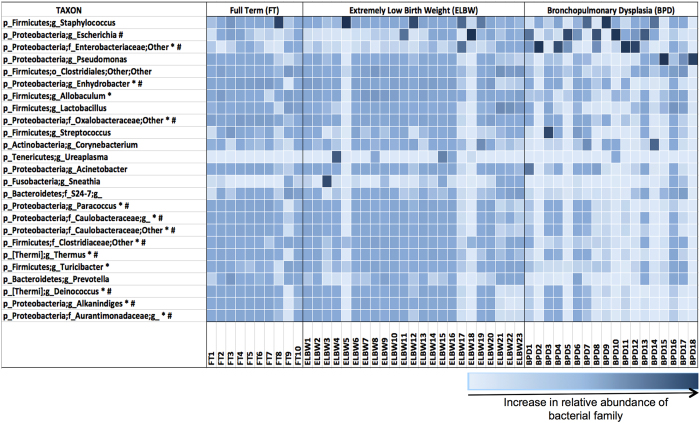
Genus level lung microbial abundance of ELBW infants, FT infants and patients with BPD. Heatmap depicting the relative abundance of the most common bacterial families at the genus level. Statistically significant difference in microbial abundance is seen between lung microbiome of ELBW and BPD infants (*) and between FT and BPD infants (#).

We were able to detect and characterize bacterial DNA in tracheal aspirates of all ELBW and FT infants soon after birth (Figs 1A and 2). All specimens resulted in amplification of the 16S rRNA used for microbiome analysis. Most mothers of premature infants received antimicrobial therapy before delivery (Table 4). On statistical analysis, there were no differences in the airway microbiome of the infants of mothers who received prenatal antibiotics versus infants of mothers who did not.

### Airway microbiome of ELBW and FT infants is similar at birth

The taxonomic analysis in this study demonstrated that the lung microbiome was similar at birth in ELBW and FT infants irrespective of gestational age (Fig. 1A,C). Both ELBW and FT infants had a predominance of *Firmicutes* and *Proteobacteria* on the first day of life, in addition to the presence of *Actinobacteria, Bacteroidetes, Tenericutes, Fusobacterium, Cyanobacteria*, and *Verrucomicrobia* (Fig. 1A). The relative abundance of bacterial phyla did not differ between ELBW and FT infants. We compared the diversity between the groups and calculated the Shannon diversity index for alpha diversity (which gives an estimate of richness, evenness, and microbial diversity within a sample). There was no difference between the Shannon alpha diversity for ELBW and FT infants (p = 0.46, t-test, Fig. 1D). As shown in the principal coordinate analysis (PCoA) plot (Fig. 1C), the BPD infant microbiomes clustered separately with a greater Unifrac distance separating them, whereas ELBW and FT microbiomes had significant overlap on the PCoA plot (p = 0.19, permanova test, Fig. 1C). *Tenericutes* including genus *Ureaplasma* were identified in the tracheal aspirates of both ELBW and FT infants (Figs 1A and 2), although its relative abundance was higher in ELBW infants.

### The microbial composition of the airway is different and less diverse in infants with BPD compared to FT and ELBW infants

Compared to newborn FT infants matched for post-menstrual age, the airway microbiome of infants after diagnosis of BPD was characterized by increased phylum *Proteobacteria* and decreased phyla *Firmicutes* and *Fusobacteria* (Fig. 1A,B). *Gamma Proteobacteria* were more abundant in BPD infants whereas *Alpha Proteobacteria* were in lower abundance in BPD infants compared to newborn ELBW and FT infants (Fig. 2). At the genus level, the most abundant *Proteobacteria* in BPD patients were *Enterobacteriaceae* (Fig. 2).

Differences in microbial composition between BPD and FT infants were confirmed by a difference in Shannon alpha diversity (p < 0.002; t-test; Fig. 1D). We used Unifrac-weighted distances for beta diversity (that are inclusive of abundance, the presence or absence of OTUs between samples, and demonstrates how different microbes are distributed among samples). We found different clustering as depicted in PCoA plots of Unifrac distances (p < 0.0001; permanova test; Fig. 1C). Differences in microbe diversity and abundance (alpha diversity p < 0.003, t-test, Fig. 1D; beta diversity p < 0.009, permanova test, Fig. 1C) were seen also between ELBW infants and infants with BPD (Figs 1 and 2).

To confirm the presence of Proteobacteria in the BPD patient samples, we also performed specific endotoxin assays, the results of which are given below.

### Airway microbiome of ELBW infants who go on to develop BPD show a consistent temporal dysbiosis

**Figure 3:**
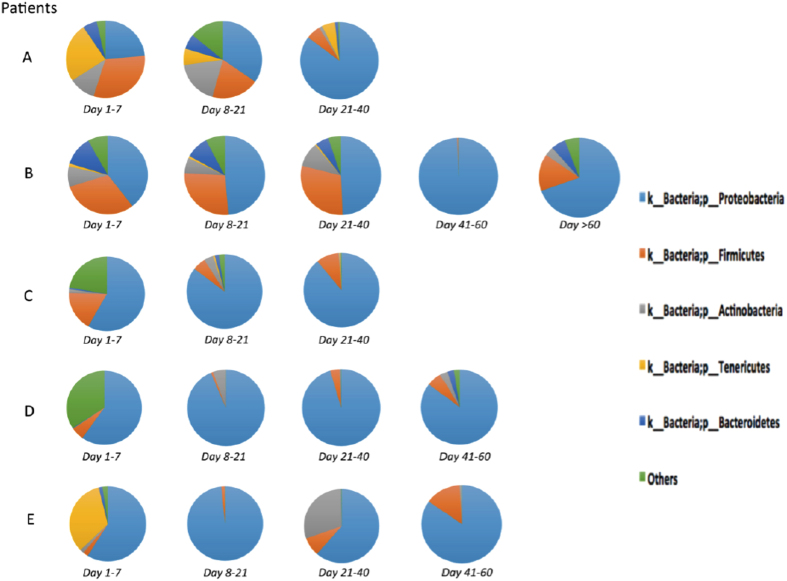
Temporal changes in airway microbiome of ELBW infants who develop BPD. Pie charts showing the changes in airway microbiome with time in 5 ELBW patients (**A–E**) from birth onwards. In all patients, a relative increase in abundance of *Proteobacteria* and a relative decrease in abundances of *Firmicutes, Actinobacteria, Tenericutes, Bacteroidetes* and *other microbes,* are seen with time.

Five extremely premature infants were followed from birth until the development of BPD and serial TA collected at various postnatal time points (Day 1–7, Day 8–21, Day 21–40, Day 41–60, Day > 60) (Fig. 3). The *first* sample (labeled day 1–7) was collected on day 1 in 4 of the 5 patients (Patient A, B, C, D), whereas in the 5^th^ patient the first sample was collected on day 5. The demographics of these infants are given in Table 5. All 5 infants, despite having multiple courses of antibiotics at various different time points, had a distinct temporal dysbiotic change with a decrease in *Firmicutes* and increase in abundance of *Proteobacteria* over time (Fig. 3).

### ELBW infants born by cesarean section (CS) and vaginal delivery have airway microbiota that do not differ at birth

Of the 23 ELBW infants in the discovery cohort, 15 (65%) were born by CS and 8 (35%) by vaginal delivery. There were no statistical differences between the airway microbiota of ELBW infants born by CS compared to the ones born by vaginal delivery (all microbial taxa: p > 0.1, t-test).

### Genus *Lactobacillus* was decreased in airway microbiota of ELBW infants with chorioamnionitis

Among our discovery cohort of 23 ELBW infants, 10 (43%) had histological chorioamnionitis. The microbiome of infants with versus without histological chorioamnionitis differed at the genus level but not at the phylum level. A decreased abundance of genus *Lactobacillus* was seen in infants exposed to chorioamnionitis as compared to infants not exposed to chorioamnionitis (p = 0.037, t-test, Fig. 3D). Among our validation cohort of 14 infants, only 2 had chorioamnionitis and hence this analysis was not possible (Table 3).

### Genus *Lactobacillus* is less abundant in the early airway microbiome of infants who later develop BPD

ELBW infants from both our discovery cohort and validation cohort were subdivided into two groups based on their pulmonary outcomes into either BPD Resistant or BPD Predisposed.

#### Discovery Cohort

**Figure 4:**
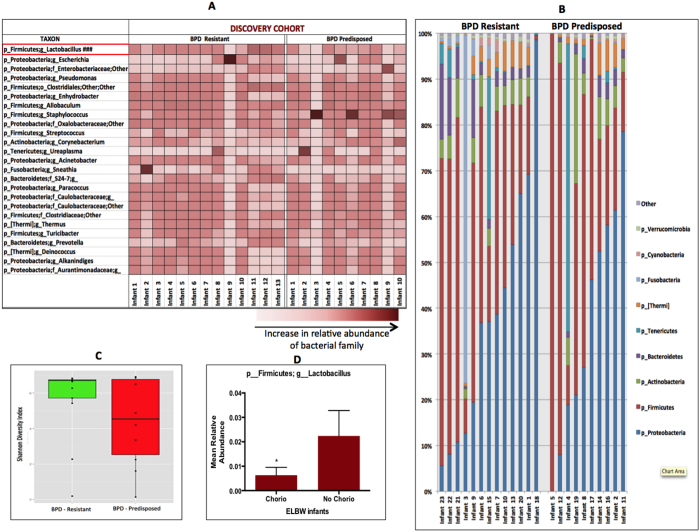
Early airway microbiome of ELBW infants and its association with development of BPD – Discovery Cohort. (**A**) Heat map showing the relative abundance of genera across day 1 samples from ELBW infants who go on to develop BPD (BPD - Predisposed) versus those who do not (BPD Resistant). Abundance of genus *Lactobacillus* (###) is reduced in BPD - Predisposed group (p = 0.05). (**B**) Phylum level distribution of lung microbiome shows no major differences between groups. (**C**) Shannon diversity index alpha diversity is not statistically different between groups (p = 0.18). (**D**) Bar graph depicting lower abundance of genus lactobacillus in infants born to mothers with chorioamnionitis.

All 23 ELBW infants survived to 36 weeks PMA and were similar in demographic characteristics (Table 2). 10 of these 23 infants went on to develop BPD (BPD Predisposed) (Fig. 4). 13 of the 23 ELBW did not develop BPD and were deemed resistant to the disease (BPD Resistant) (Fig. 4). The relative phylum level microbial abundance (Fig. 4A,B) and alpha diversity (p = 0.18, Fig. 4C) were not statistically different between the BPD Resistant and BPD Predisposed groups. However at the genus level, genus *Lactobacillus* was less abundant in BPD Predisposed infants at birth, compared to the BPD Resistant infants (p < 0.05, t-test; Fig. 4A).

#### Validation Cohort

**Figure 5:**
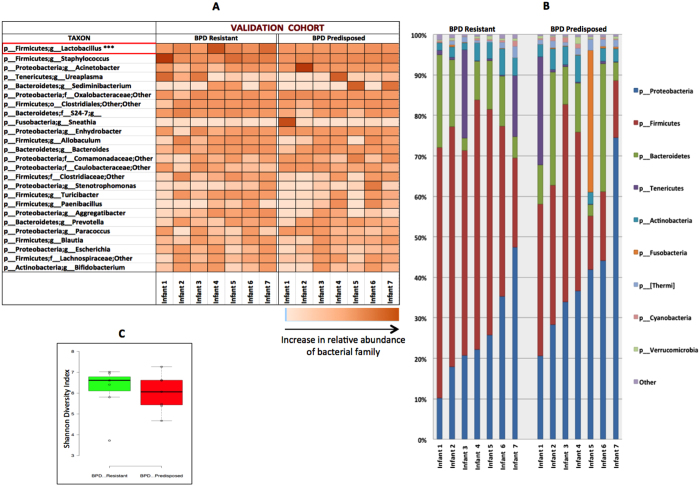
Early airway microbiome of ELBW infants and its association with development of BPD – Validation Cohort. (**A**) Heat map showing the relative abundance of genera across day 1 samples from ELBW infants who go on to develop BPD (BPD Predisposed) versus those who do not (BPD Resistant). Abundance of genus *Lactobacillus* (***) is reduced in BPD Predisposed group (p = 0.04). (**B**) Phylum level distribution of lung microbiome shows no major differences between groups. (**C**) Shannon diversity index shows no difference in alpha diversity between groups (p > 0.1).

ELBW infants from our validation cohort were similarly subdivided into two groups based on their pulmonary outcomes – BPD Resistant (7 infants) and BPD Predisposed (7 infants). Both groups were similar in demographic characteristics (Table 3). Consistent with the findings of our discovery cohort, *Lactobacillus* was less abundant at the genus level in BPD Predisposed infants at birth compared to the BPD Resistant (p < 0.04, t-test; Fig. 5A). The relative phylum level microbial abundance (Fig. 5A,B) and diversity (p > 0.1, Fig. 5C) were not statistically different between the BPD Resistant or BPD Predisposed groups.

**Figure 6:**
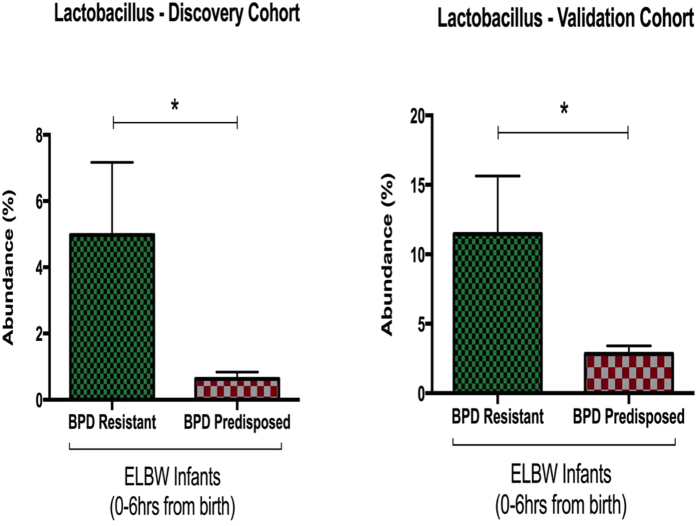
Early airway *Lactobacillus* abundance in discovery and validation cohorts. Bar graphs depicting the relative abundance of *Lactobacillus* in ELBW infants at birth. In both the discovery cohort and the validation cohort the relative abundance of *Lactobacillus* is significantly higher in the BPD Resistant infants compared to BPD Predisposed infants (p < 0.05).

Figure 6 demonstrates the relative difference in *Lactobacillus*, in both *discovery* and *validation* cohorts.

### Endotoxin levels are increased in airways of infants with established BPD

**Figure 7:**
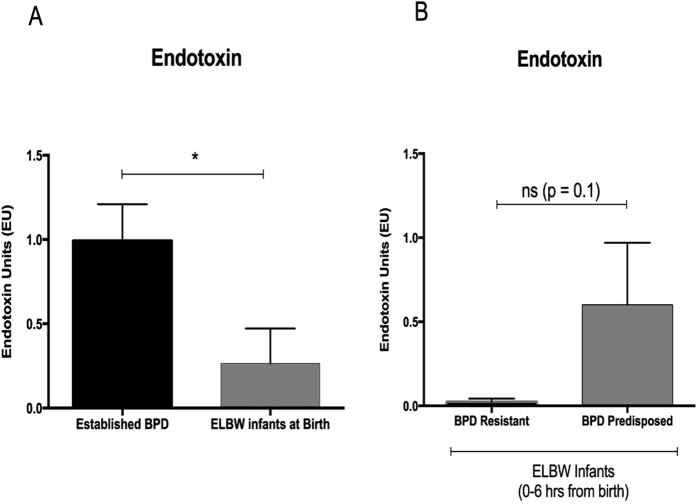
Limulus amebocyte lysate chromogenic *endotoxin* levels in tracheal aspirates of infant. (**A**) Endotoxin concentrations are increased in tracheal aspirate samples collected from infants with BPD compared to infants at birth (p < 0.05). (**B**) No difference was seen in endotoxin amounts in airways of BPD Resistant and BPD Predisposed ELBW infants at birth (p = 0.1).

Using a highly specific limulus amebocyte lysate (LAL) endotoxin assay we determined endotoxin (proteobacterial products) concentrations in TA. Endotoxin expression was significant increased in infants with established BPD compared to infants at birth (Fig. 7A, p < 0.05). This corresponds to our airway microbiome analysis finding of increased proteobacteria in infants with established BPD, compared to ELBW or FT infants at birth. At birth, endotoxin concentrations in airways of BPD Resistant and BPD Predisposed ELBW infants showed a statistically non-significant trend towards increase in BPD Predisposed infants, (Fig. 7B, p = 0.1). In addition, no difference was seen in endotoxin levels in ELBW infants versus FT infants at birth (p > 0.1, data not shown).

## Discussion

In this study, we evaluated the airway microbiome of extremely preterm and term infants soon after birth and in preterm infants with established BPD. A major novel finding was that the airways have a diverse microbiome even at birth regardless of gestational age. To date, it has been considered that infant mucosal surfaces are populated by skin, vaginal, and intestinal microbes derived from the mother^9^. As it is commonly believed that colonization of neonates originates in the birth canal, we were surprised to find that the airway microbiome of vaginally delivered and caesarean section-delivered neonates were similar, which suggests that the microbial DNA in the airways is probably transplacentally derived, consistent with reports that the placenta has a rich microbiome^10^. We also found that airway microbiota in infants with BPD has decreased diversity and is very different from that of either preterm infants soon after birth, or full term infants at a similar post-menstrual age. Our observations indicate that the early airway microbiota is associated with the development of BPD in ELBW infants and the airway microbiome shows consistent changes over time from birth to the development of BPD.

In the absence of direct access to the newborn lung tissue and due to the inability to obtain broncheoalveolar lavage fluid in newborn infants, tracheal aspirates are a surrogate for evaluation of the processes in the lower airways and distal lungs. To date, two studies have utilized culture independent methods to detect airway organisms in preterm infants. In a study of 10 infants, Mourani *et al*.^11^ demonstrated that airways of premature infants are not sterile. In their study, *Staphylococcus, Ureaplasma parvum*, and *Ureaplasma urealyticum* were the most frequently identified dominant organisms, among other identified organisms like *Pseudomonas, Enterococcus*, and *Escherichia*. Lohmann *et al*.^12^ studied the respiratory microbiome of 25 preterm infants but did not evaluate the microbiota of normal term infants. Our study provides a more detailed comparison of the respiratory microbiome of ELBW, FT and BPD infants. The tight gestational age criteria in our ELBW group (mean gestation of 24.3 ± 1.5 weeks) helped us study the infants who are at the highest risk of developing BPD. In our cohort, most mothersof ELBW infants received prenatal antimicrobial therapy close to delivery. As it is routine clinical practice to treat mothers in preterm labor with antimicrobials, it is difficult to generate an adequate sample size to determine the effect of antimicrobial therapy on the neonatal airway microbiome as there are very few infants who are not exposed.

A strength of our study was the sterile collection of tracheal aspirate samples from intubated ELBW and FT infants at or within six hours of birth, thus reducing the chances of contamination or postnatal colonization. Only samples with >1000 sequence reads were utilized for the qualitative analysis, thus further reducing the chances of erroneous results. Samples with low bacterial biomass were discarded. The increase in *Proteobacteria* in infants established BPD was marked by a corresponding increase in endotoxin levels, which adds validity to our microbiome analysis.

In this study we employed genomic approaches rather than culture, and utilized separate development and validation cohorts to explore the predictive role of airway microbiome at birth for the future development of BPD. A central finding was the decreased *Lactobacillus* abundance in airway microbiome of infants born to mothers with chorioamnionitis. Gritz and Bhandari^13^ have recently highlighted the importance of *Lactobacillus spp*. in preterm infants. Also, as chorioamnionitis is an independently associated risk factor for BPD^14^, this finding could be important in defining the association of chorioamnionitis and BPD. We also found decreased *Lactobacillus* abundance at birth in the airways of BPD-Predisposed ELBW infants as compared to BPD-Resistant infants. This finding is congruent with findings of Lynch *et al*.^15^,^16^,^17^, who have reported a beneficial role of lactobacillus in other airway diseases. Studies in mice have shown that intranasal administration of lactobacilli may be more potent than intragastric application in reducing allergic airway inflammation, possibly linked to an increase in T-regulatory cells in the lungs^18^. Other investigators have shown that higher bacterial abundance in non-specific-pathogen-free mice correlated with more and smaller size alveoli indicating better lung development, which was corroborated by transplanting *Lactobacillus spp.* into germ-free mice, which responded by improvement in alveolar development^19^. We have recently shown that fecal microbiota in preterm infants may vary by center^20^, and it is possible that there is center variation in airway microbiota as well, which may lead to center variation in short-term as well as longer-term pulmonary outcomes. Hence, we validated our findings with patient samples from a different center. Relative decreased *Lactobacillus* abundance at birth in airway microbiome was found to be predictive of BPD in the validation cohort as well. Our results indicate that the respiratory microbiome at birth could potentially be used for prognosis, and may potentially be modulated perhaps using “respiratory probiotics” for therapeutic interventions in preterm lung disease. Although it is known that mode of delivery can shape the acquisition and structure of the skin microbiota in newborns^9^, our study found no differences between the respiratory microbiome of ELBW infants born by cesarean section versus those born by vaginal delivery. This finding indicates that the airway microbiome is probably established well before delivery (as it is diverse and similar in both extremely preterm and term infants), and is potentially through a transplacental passage of bacterial products. Aagard *et al*.^10^ have shown that the placental microbiome is very diverse, resembles the oral microbiome, and is similar to the airway microbiome in this study. Stout *et al*.^21^ have also demonstrated gram positive and negative intracellular bacteria in the basal plates of 27% of all placentas, even though it is highly probable that visual identification in a few selected high power fields is likely to be a marked underestimate. We speculate that the biologic rationale for why the placental microbiome resembles the oral microbiome^10^ and the neonatal airway microbiome is because a hematogenous transfer of oral microbes to the placenta^10^,^22^,^23^,^24^,^25^ followed by transplacental transfer of placental microbiota ensures that the early airway microbiome and its associated pathogen-associated molecular patterns can initiate establishment of respiratory innate immunity before respiratory pathogens can establish a niche. Alternatively there could be a local microbial transfer from placenta to the amniotic fluid, which the fetal lungs acquire. Multiple studies utilizing culture and culture-independent have now confirmed that various bacterial species are detected in amniotic fluid^26^. The amniotic fluid microbiota and the airway microbiota could have a common origin from the placenta, and the level of microbial cross-transfer between these pools needs to be determined.

Although all attempts were made to collect TA specimens in a sterile fashion, there is a possibility that contamination could have occurred as inhaled microbiota could contribute to the airway microbiome^27^. This could explain the increased Genus *Staphylococcus* abundance at birth in the airways of BPD - Predisposed ELBW infants in our discovery cohort, which was not validated in the samples from our second site. Also, TAs might not represent the true microbiome of the distal lungs and could contain oral microbiota or aspirated gastric microbiota in addition to airway microbiota. Hence we employed our research strategy of very early (at birth or within 6 hours) collection of TA samples, when none of the infants were feeding. In addition we used samples with only >1000 sequence reads. A limitation of our study is the absence of “normal” full term controls to compare with diseased group, as normal infants do not get intubated. Although our FT infant cohort might not be the perfect controls, they were the only and the closest available options. Another limitation is the qualitative nature of our study, as we have not performed individual bacterial quantifications.

Serial microbiome analysis from the same patient may yield useful information on temporal changes leading to BPD. Hence we collected TA samples from extremely preterm infants in a serial fashion, and found consistent temporal changes in the airway microbiome from birth until the development of BPD. These infants were of the smallest viable gestational ages from 22–24 weeks, and hence were the most prone to develop BPD. All five infants had similar temporal changes in the airway microbiota – that of increasing abundance of *Proteobacteria* and decreased *Firmicutes* in airway microbiome over time, despite multiple courses of antibiotics at different time points. Genus *Lactobacillus* has been known to have strong anti-inflammatory properties^28^,^29^,^30^,^31^,^32^,^33^, and has been shown to regulate alveolar development in animal models^19^. Hence, the temporal microbial dysbiosis consisting of the increase in *Proteobacteria* and decrease in *Firmicutes* such as *Lactobacillus* may contribute to the airway inflammation associated with BPD and resulting impairment in lung development. The serial tracheal sample findings in our study do show that the initial lung microbiome in infants tends to evolve over time. It is likely that the postnatal changes in the airway microbiome are driven by exposure to environmental microbes, and is modulated by antimicrobial therapy to the infant.

Further understanding of the role of the complex airway microbiome in preterm infants requires investigation of the interactions among genes of the microbiota and host. There is a possibility that the microbiome may have beneficial cross-talk with the host preterm lung and the reduced diversity of microbes resulting from intensive care interventions makes the host lung more susceptible for injury and inflammation. The lung microbiome, similar that of other compartments, may be manipulated to correct dysbiosis and restore “healthy” microbial communities via use of probiotics, prebiotics or antibiotics. Early life gut microbial alterations include changes in the production of microbial-derived metabolites, and a similar phenomenon may happen in the lungs^34^. Hence, future in-depth studies of the fetal pulmonary microbiome are warranted and are ongoing. In addition, further research on the gut–lung axis and the potential role of extra-pulmonary microbes in the development of respiratory disease needs to be explored.

## Methods

We conducted a prospective observational cohort study between October 2014 and March 2015 at the University of Alabama at Birmingham (UAB) Regional Neonatal Intensive Care Unit. The Institutional Review Board of University of Alabama at Birmingham approved the human subject protocol and granted waiver of consent status as samples were obtained from routine clinical care and were handled in a completely de-identified manner. The methods were carried out in accordance with the approved guidelines. Further details are in the online data supplement.

### Patient population

#### Discovery Cohort (UAB)

Inborn and outborn ELBW and FT infants who underwent endotracheal intubation and mechanical ventilation at birth (in the first 6 hours of life) were included. In addition, infants diagnosed with moderate or severe BPD at 36 weeks’ PMA defined using the physiologic definition^35^ were included. Samples from a total of 150 infants were collected but only 51 were utilized for analysis presented in this manuscript based on strict inclusion criteria (23 ELBW infants and 10 FT infants – samples obtained at birth or within 6 hours of birth at the time of intubation; 18 infants with established BPD - samples obtained at 36 weeks PMA at the time of ETT change; samples with only >1000 bacterial sequence reads were used). All 10 full term infants enrolled, were intubated at or within 6 hours of birth due to either surgical indications (congenital heart disease, abdominal wall defect) or due to perinatal depression (with no signs of meconium aspiration syndrome). Sample size calculations could not be performed as no baseline data in this population was available, this being the first study of its kind in the field, but statistical power was established using standard statistical methods as described later.

No specific exclusions were made. Chorioamnionitis was determined by placental histopathology and all mothers with chorioamnionitis were administered antibiotics prenatally.

#### Validation Cohort (Philadelphia)

We utilized patient samples from Philadelphia as a validation cohort to confirm our data. TA samples from Inborn and outborn ELBW infants who underwent endotracheal intubation and mechanical ventilation at birth were included. These samples were collected as part of ongoing studies^36^,^37^,^38^.

### Sample collection

Tracheal aspirate (TA) specimens were obtained from patients at the time of intubation in the first hours of life and thereafter whenever tracheal suctioning was clinically indicated, per unit protocol. Samples were obtained after ensuring that the infant was adequately oxygenated. The protocol for TA collection involved instillation of 1 mL sterile isotonic saline into the infant’s endotracheal tube, manual bagging through the endotracheal tube for three breaths, and suctioning of the fluid into a sterile mucus trap. Samples were stored frozen in −80^o^C until further processing. A similar protocol was employed at both study sites.

### Isolation of microbial DNA, creation of 16S V4 amplicon library and DNA sequencing

Microbial genomic DNA was isolated and PCR was used with unique bar coded primers to amplify the V4 region of the 16S rRNA gene to create an “amplicon library” from individual samples^39^,^40^. The PCR products were sequenced using the NextGen sequencing Illumina MiSeq platform^39^. The sequence data covered the 16S rRNA V4 region with a PCR product length of ~255 bases and 250 base paired-end reads. Sequences were grouped into operational taxonomic units (OTUs). Only samples with >1000 reads were included. Alpha diversity value (within sample diversity) was calculated using Shannon’s indez^41^. Difference in alpha diversity amongst samples was calculated using t-tests. Unifrac analysis was used to determine relationships between different samples (beta diversity)^42^. Differences in beta diversity among different groups were measured using permanova test. Principal coordinates analysis (PCoA) was used visualize the dissimilarity between all the samples. 3D PCoA plots were generated using EMPEROR^43^. All raw data files have been uploaded online on Sequence Read Archive (SRA - SUB1512169).

### Clinical data collection

Data collection was performed via electronic medical record review at time of enrollment and during remainder of hospitalization. Chorioamnionitis was determined by histopathology, BPD at 36 weeks’ post-menstrual age was defined by the physiologic definition^35^, and late sepsis was defined as a positive blood culture after 72 h of life.

## Additional Information

**How to cite this article**: Lal, C. V. *et al*. The Airway Microbiome at Birth. *Sci. Rep.* **6**, 31023; doi: 10.1038/srep31023 (2016).

## Supplementary Material

Supplementary Information

## Figures and Tables

**Table 1:** Demographic characteristics of ELBW, FT and BPD infants.

	Full term infants, sampled on day 1 after birth **(FT)**	ELBW infants, sampled on day 1 after birth **(ELBW)**	Infants with established bronchopulmonary dysplasia **(BPD)**
Study population, *n*	10	23	18
Postmenstrual age tracheal aspirate, weeks ± SD	38.3 ± 2.2	24.5 ± 0.2	37.6 ± 1.5
Gestational age, weeks ± SD	38.2 ± 2.2	24.3 ± 1.5	25.3 ± 2.1
Birth weight, grams ± SD	3334 ± 521	645 ± 163	731 ± 287
Male sex, no. (%)	8 (80)	8 (35)	9 (50)
Race			
Black, no. (%)	5 (50)	17 (74)	12 (67)
White, no. (%)	5 (50)	6 (26)	6 (33)
Antenatal corticosteroids, no. (%)	1 (10)	22 (96)	16 (89)
Chorioamnionitis, no. (%)	2 (20)	10 (43)	9 (50)
Rupture of membranes > 18 hours, no. (%)	3 (30)	4 (17)	5 (28)
Cesarean section, no. (%)	4 (40)	15 (65)	12 (67)
Pre-eclampsia, no. (%)	1 (10)	13 (57)	5 (28)
Intrauterine growth restriction, no. (%)	0 (0)	10 (43)	6 (33)
Intubation in delivery room, no. (%)	3 (30)	10 (43)	12 (67)
Respiratory distress syndrome, no. (%)	2 (20)	22 (96)	18 (100)
Treatment with surfactant, no. (%)	2 (20)	21 (91)	18 (100)
High frequency ventilation, no. (%)	1 (10)	7 (26)	7 (39)
Ventilator days, mean ± SD	6 ± 7	32 ± 38	77 ± 49
CPAP days, mean ± SD	1 ± 2	21 ± 19	54 ± 38
Oxygen days, mean ± SD	9 ± 11	92 ± 58	225 ± 92
Died, no. (%)	0 (0)	1 (4)	7 (39)
Bronchopulmonary dysplasia, no. (%)	0 (0)	10 (43)	18 (100)
Pulmonary hemorrhage, no. (%)	0 (0)	5 (22)	2 (11)
Symptomatic patent ductus arteriosus, no. (%)	0 (0)	9 (39)	9 (50)
Sepsis, no. (%)	0 (0)	8 (35)	13 (72)
Intracranial hemorrhage ≥ grade 3, no. (%)	0 (0)	2 (9)	1 (6)
Retinopathy of prematurity ≥ stage 3, no. (%)	0 (0)	2 (9)	4 (22)
Necrotizing enterocolitis ≥ stage 2, no. (%)	0 (0)	5 (22)	4 (22)

**Table 2:** Discovery Cohort – Demographics.

	Discovery cohort	
	BPD - Resistant	BPD - Predisposed
Study population, *n*	13	10
Gestational age, weeks ± SD	24.8 ± 1.7	23.5 ± 0.7
Birth weight, grams ± SD *	686 ± 140	564 ± 93
Male sex, no. (%)	4 (31)	4 (40)
Race		
Black, no. (%)	10 (77)	7 (70)
White, no. (%)	3 (23)	3 (30)
Antenatal corticosteroids, no. (%)	12 (92)	10 (100)
Chorioamnionitis, no. (%)	6 (46)	4 (40)
Rupture of membranes > 18 hours, no. (%)	3 (23)	1 (10)
Cesarean section, no. (%)	8 (62)	7 (70)
Pre-eclampsia, no. (%)	7 (54)	6 (60)
Intrauterine growth restriction, no. (%)	4 (31)	6 (60)
Intubation in delivery room, no. (%)	5 (38)	5 (50)
Respiratory distress syndrome, no. (%)	12 (92)	10 (100)
Treatment with surfactant, no. (%)	11 (85)	10 (100)
High frequency ventilation, no. (%)	2 (15)	5 (50)
Ventilator days, mean ± SD *	13 ± 18	58 ± 43
CPAP days, mean ± SD	14 ± 14	29 ± 23
Oxygen days, mean ± SD *	51 ± 39	145 ± 26
Died, no. (%)	0 (0)	0 (0)
Pulmonary hemorrhage, no. (%)	2 (15)	3 (30)
Symptomatic patent ductus arteriosus, no. (%)	6 (46)	3 (30)
Sepsis, no. (%)	4 (31)	4 (40)
Intracranial hemorrhage ≥ grade 3, no. (%)	2 (15)	0 (0)
Retinopathy of prematurity ≥ stage 3, no. (%)	0 (0)	2 (20)
Necrotizing enterocolitis ≥ stage 2, no. (%)	1 (8)	4 (40)

**Table 3:** Validation Cohort – Demographics.

	Validation cohort	
	(BPD - Resistant)	(BPD - Predisposed)
Study population, *n*	7	7
Gestational age, weeks ± SD	25.1 ± 0.7	24.8 ± 1.7
Birth weight, grams ± SD	700 ± 340	686 ± 140
Male sex, no. (%)	5 (71)	4 (57)

**Table 4:** Maternal antibiotic administration prior to birth in ELBW infants.

Patient	Maternal Antibiotics (within 72 hrs prior to birth)	Type of Antibiotics
BPD Predisposed		
1	No	
2	Yes	ampicillin, amoxicillin, gentamicin
3	No	
4	No	
5	Yes	cefazolin
6	Yes	ampicillin
7	Yes	amphotericin B
8	Yes	acyclovir
9	Yes	ampicillin, gentamicin
10	Yes	cefazolin
BPD Resistant		
1	Yes	ampicillin
2	Yes	amphotericin B, azithromycin, clindamycin, gentamicin
3	Yes	cefazolin
4	No	
5	Yes	ampicillin, amoxicillin, azithromycin
6	Yes	ampicillin
7	Yes	ampicillin, amoxicillin, azithromycin
8	Yes	ampicillin
9	Yes	ampicillin
10	Yes	ampicillin
11	Yes	ampicillin, cefepime, vancomycin, acyclovir
12	No	
13	Yes	acyclovir

**Table 5:** Demographic characteristics of 5 ELBW infants who later develop BPD.

Patient	A	B	C	D	E
Gestational age, weeks	23	23	23	22	24
Birth weight, grams	510	660	640	460	520
Sex	Female	Male	Male	Female	Female
Race	Caucasian	African American	Caucasian	Caucasian	African American
Antenatal corticosteroids	Y	Y	Y	N	Y
Chorioamnionitis	N	Y	Y	Y	Y
Rupture of membranes >18 hours	N	Y	N	N	Y
Pre-eclampsia	N	N	N	N	N
Intrauterine growth restriction	Y	N	N	N	N
Apgar - 1 minute	5	3	2	—	1
Apgar - 5 minutes	8	8	2	—	3
Intubation in delivery room	Y	N	Y	Y	Y
Respiratory distress syndrome	Y	Y	Y	Y	Y
Treatment with surfactant	Y	Y	Y	Y	Y
High frequency ventilation	N	N	N	Y	Y
Pulmonary hemorrhage	N	N	N	N	Y
Symptomatic patent ductus arteriosus	Y	N	Y	N	N
Sepsis	Y	Y	N	N	N
Intracranial hemorrhage ≥ grade 3	N	N	N	Y	N
Retinopathy of prematurity ≥ stage 3	Y	N	N	N	N
Necrotizing enterocolitis ≥ stage 2	Y	N	N	N	N
