# Supplementary material for: The Airway Microbiome at Birth
Source: Sci Rep. 2016 Aug 4;6:31023. doi: 10.1038/srep31023 (PMC4973241; doi:10.1038/srep31023)
Supplement: Supplementary Information [file srep31023-s1.doc]

**ONLINE DATA SUPPLEMENT**

**The Airway Microbiome at Birth**

Charitharth Vivek Lal^1, 2, 3*^, Colm Travers^1^, Zubair H. Aghai^4^, Peter Eipers^6^, Tamas Jilling^1, 2^, Brian Halloran^1, 2^, Waldemar A. Carlo^1^, Jordan Keeley^1^, Gabriel Rezonzew^1^, Ranjit Kumar^5^, Casey Morrow^6^, Vineet Bhandari^7^, Namasivayam Ambalavanan^1, 2, 5^

**Affiliations:**

^1^Division of Neonatology, Department of Pediatrics, University of Alabama at Birmingham, AL;

^2^Translational Research in Normal and Disordered Development Program (TReNDD) and ^3^Program in Protease and Matrix Biology, University of Alabama at Birmingham, AL; ^4^Department of Pediatrics, Thomas Jefferson University/Nemours, Philadelphia, PA;

^5^Center for Clinical and Translational Sciences, University of Alabama at Birmingham, AL; ^6^Department of Cell, Developmental and Integrative Biology, University of Alabama at Birmingham, AL;

^7^Department of Pediatrics, Drexel University College of Medicine, Philadelphia, PA

**Address correspondence to*:**

Charitharth Vivek Lal, M.D

Division of Neonatology, Department of Pediatrics,

University of Alabama at Birmingham,

176F Suite 9380, Women and Infants Center,

619 South 19th Street, Birmingham, AL 35249-7335

**Tel:**  (205) 934 4680 **Fax**: (205) 934-3100 **Email:** [clal@peds.uab.edu](mailto:clal@peds.uab.edu)

**Online Supplement:**

Methods:

**Isolation of Microbial DNA and Creation of 16S V4 amplicon Library:**

Microbial genomic DNA was isolated using the Fecal DNA isolation kit from Zymo Research (catalog # D6010; Zymo Research Corporation, Irvine, CA). Once the sample DNA was prepared, PCR was used with unique bar coded primers to amplify the V4 region of the 16S rRNA gene to create an “amplicon library” from individual samples . Following PCR, the entire PCR reaction was electrophoresed on a 1.0% agarose/Tris-borate-EDTA gel.

**DNA sequencing:**

The PCR products were sequenced using NextGen sequencing Illumina MiSeq platform [^39^](#_ENREF_39). The MiSeq is a single flowcell, single lane instrument that can generate approximately 9 Gb of sequence data from a paired end 250 bp run . We used the paired 250 base pair end kits from Illumina for the V4 region in the microbiome analysis. The samples were first quantitated using Pico Green, adjusted to a concentration of 4 nM then used for sequencing on the Illumina MiSeq[^40^](#_ENREF_40). Fastq conversion of the raw data files was performed following de-multiplexing. Quality control of the fastq files was performed then subject to quality assessment and filtering using the FASTX toolkit (FASTX). The remainder of the steps was performed with the Quantitative Insight into Microbial Ecology (QIIME) suite, version 1.7.

**Bioinformatics and Statistical Analysis:**

The sequence data covered the 16S rRNA V4 region with a PCR product length of ~255 bases and 250 base paired-end reads. Since the overlap between fragments was approximately 245 bases, the information from both ends of the paired reads was merged to generate a single high quality read using the module “fastq_mergepairs” of USEARCH [^47^](#_ENREF_47). Read pairs with an overlap of less than 50 bases or with too many mismatches (> 20) in the overlapping region were discarded. Chimeric sequences were also filtered using the “identify_chimeric_seqs.py” module of USEARCH [^47^](#_ENREF_47). Overall read quality was assessed before and after filtering using FASTQC (FASTQC). The QIIME data analysis package was used for subsequent 16S rRNA data analysis. Sequences were grouped into operational taxonomic units (OTUs) using the clustering program UCLUST at a similarity threshold of 0.97% [^47^](#_ENREF_47). The Ribosomal Database Program (RDP) classifier was used to make taxonomic assignments (to the species level) for all OTUs at confidence threshold of 60% (0.6)[^48^](#_ENREF_48). The RDP classifier was trained using the Greengenes (v13_8) 16S rRNA database[^49^](#_ENREF_49). The resulting OTU table included all OTUs, their taxonomic identification, and abundance information. OTUs whose average abundance was less than 0.0005% were filtered out. OTUs were then grouped together to summarize taxon abundance at different hierarchical levels of classification (e.g. phylum, class, order, family, genus, and species). These taxonomy tables were also used to generate bar charts of taxon abundance. Multiple sequence alignment of OTUs was performed with PyNAST [^50^](#_ENREF_50). Alpha diversity (within sample diversity) was calculated using a variety of diversity metrics including Shannon’s, Chao1, and Simpson, as implemented in QIIME[^41^](#_ENREF_41). Beta diversity (between sample diversity) among different samples was measured using Unifrac analysis, t tests[^42^](#_ENREF_42). Principal coordinates analysis (PCoA) was performed by QIIME to visualize the dissimilarity matrix (beta-diversity) between all the samples, such that samples which are more similar are closer in space than samples that are more divergent. 3D PCoA plots were generated using EMPEROR[^43^](#_ENREF_43). A heatmap with the top 50 most highly abundant taxa across all samples was generated using the “heatmap.2” function in R package (available at [http://CRAN.R-project.org/package=gplots](http://cran.r-project.org/package=gplots)).

**Endotoxin Assay:** The endotoxin concentration in samples was measured using the Pierce *Limulus* Amebocyte Lysate (LAL) assay Chromogenic Endotoxin Quantitation Kit (Pierce: 88282) via a chromogenic signal generated in the presence of endotoxins. A standard curve was created using the *E. coli* endotoxin standard included with each kit to calculate endotoxin levels as low as 0.1 EU/mL, where one endotoxin unit/mL (EU/mL) equals approximately 0.1ng endotoxin/mL of solution.
